# Evaluating the relationship between change in performance on training tasks and on untrained outcomes

**DOI:** 10.3389/fnhum.2014.00617

**Published:** 2014-08-13

**Authors:** Elizabeth M. Zelinski, Kelly D. Peters, Shoshana Hindin, Kevin T. Petway, Robert F. Kennison

**Affiliations:** ^1^Zelinski Laboratory, Center for Digital Aging, Davis School of Gerontology, University of Southern CaliforniaLos Angeles, CA, USA; ^2^Psychology Department, University of Southern CaliforniaLos Angeles, CA, USA; ^3^Psychology Department, California State UniversityLos Angeles, CA, USA

**Keywords:** cognitive training, aging, untrained outcomes, multimodal training

## Abstract

Training interventions for older adults are designed to remediate performance on trained tasks and to generalize, or transfer, to untrained tasks. Evidence for transfer is typically based on the trained group showing greater improvement than controls on untrained tasks, or on a correlation between gains in training and in transfer tasks. However, this ignores potential correlational relationships between trained and untrained tasks that exist before training. By accounting for crossed (trained and untrained) and lagged (pre-training and post-training) and cross-lagged relationships between trained and untrained scores in structural equation models, the training-transfer gain relationship can be independently estimated. Transfer is confirmed if only the trained but not control participants' gain correlation is significant. Modeling data from the Improvement in Memory with Plasticity-based Adaptive Cognitive Training (IMPACT) study (Smith et al., 2009), transfer from speeded auditory discrimination and syllable span to list and text memory and to working memory was demonstrated in 487 adults aged 65–93. Evaluation of age, sex, and education on pretest scores and on change did not alter this. The overlap of the training with transfer measures was also investigated to evaluate the hypothesis that performance gains in a non-verbal speeded auditory discrimination task may be associated with gains on fewer tasks than gains in a verbal working memory task. Gains in speeded processing were associated with gains on one list memory measure. Syllable span gains were associated with improvement in difficult list recall, story recall, and working memory factor scores. Findings confirmed that more overlap with task demands was associated with gains to more of the tasks assessed, suggesting that transfer effects are related to task overlap in multimodal training.

## Introduction

Longitudinal declines in many cognitive processes, including memory, attention, working memory, and speed of processing, are normative in aging (e.g., Zelinski et al., 2011a). This has led to concerns that declines may negatively impact quality of life and increase the risk of losing independence, as cognition plays an important role in many activities of daily living including financial management (e.g., Jobe et al., 2001). At the same time, it has become increasingly clear that individual differences in healthy older adults' cognitive performance is associated with a wide range of potentially enriching experiences, including education, healthy lifestyle practices, engagement in cognitively challenging activities, social involvement, avoidance of stress, and positive attitudes that promote psychological well-being (Hertzog et al., 2009). Interventions to enhance cognition have also shown benefits; many of these involve training on tasks thought to benefit processes that decline with aging. An important indicator of the effectiveness of interventions designed to improve cognitive performance in older adults is whether training benefits generalize to tasks or cognitive activities that were not trained (e.g., Jobe et al., 2001). It is well established that training of specific strategies, such as mnemonics, does not produce transfer in older adults (e.g., Park et al., 2007). This approach to training holds little promise for reducing risk of decline or even supporting the maintenance of cognitive ability, possibly because older adults often do not apply strategies to new tasks. This may occur because older people experience difficulties in engaging such strategies (Zelinski, 2009), have greater willingness to use suboptimal strategies (Hertzog et al., 2007), or have poor memory self-concept (West et al., 2008).

However, extended practice of tasks such as dual-tasking or N-back, can transfer to untrained tasks (Zelinski, 2009). Game play that involves repetitive practice of cognitive skills that involve multitasking also can produce transfer (e.g., Basak et al., 2008; Anguera et al., 2013). A recent meta-analysis directly evaluated effects of extended practice cognitive training on untrained tasks. These interventions significantly improved older adults' performance on untrained cognitive tasks, with an estimated mean effect size of 0.32 after accounting for practice in the experimental and control groups (Hindin and Zelinski, 2012). All of the 25 extended practice studies in the meta-analysis evaluated improvements in untrained outcomes by comparing pre-post differences between experimental and control groups. None examined how individuals' performance was affected by training. Yet if transfer has occurred, those in the experimental group who gain more on the training task should improve more on the untrained task because the training should generalize to other tasks with common components (e.g., Persson et al., 2007; Lövdén et al., 2010) or at least the same task-specific demands (e.g., Buschkuehl et al., 2012). Several studies published subsequently to the Hindin and Zelinski meta-analysis have examined correlations between improvements on trained and untrained tasks in older adults, reporting significant correlations in the experimental group (e.g., Anguera et al., 2013; Stepankova et al., 2014).

McArdle and Prindle (2008) suggested that it is necessary to test for transfer with a more sophisticated modeling approach than the use of *t*-tests, ANOVA, or bivariate correlations. They argued that if trained and untrained tasks invoke similar constructs, these should be correlated at baseline as well as after training. This suggests that in order to assess transfer, existing relationships between performance on trained and untrained tasks at baseline should be accounted for, so that the independent relationship between baseline and posttest training and transfer task performance relationships can be ascertained. Relationships between the initial baseline and posttraining scores should also be accounted for, as individual differences in the construct measured may be related to performance gains (see also von Bastian et al., 2013). Therefore, the strongest test of whether training produces transfer is that those who received the training intervention show a significantly stronger relationship between changes in trained and untrained task performance after training than those in the control group after all other possible relationships between trained and untrained tasks prior to, as well as subsequent to, training in each group have been accounted for. It would also be expected that demographic covariates should not affect transfer if a clear interpretation of training benefits is to be made. Otherwise, interactions between the characteristics of participants and training might confound transfer.

McArdle and Prindle (2008) evaluated a series of structural equation models accounting for relationships between near (trained) and far (untrained) cognitive tasks that compared 699 participants trained over 10 h to improve reasoning with 698 members of a no-contact control group. Data were from the initial phase of the Advanced Cognitive Training for Independent and Vital Elderly (ACTIVE) trial (Ball et al., 2002), a randomized controlled single-blind study of three interventions examining whether older adults' cognitive abilities and everyday functioning could be improved over 2 years. The trained group had a higher latent change mean than the untrained group on the reasoning measures, as they had in the study, showing that training improved performance on the trained measure. The models also indicated that at baseline, relationships were significant and positive between the trained and untrained measures. There was also a significant and positive relationship between the trained and untrained latent change measures, but this relationship did not vary differentially for the trained and control group participants. Thus, this study showed no relationship between change in training and in transfer in the experimental group participants. However, no group effects of transfer had been observed in the main study (Ball et al., 2002), and the elegant structural analysis of McArdle and Prindle did not produce any new findings to support the existence of training-related transfer in the trained group. The present analyses extended the modeling approach of McArdle and Prindle to a different dataset that had produced transfer effects at the group level for the trained participants.

### Hypotheses

Data were from The Improvement in Memory with Plasticity-based Adaptive Cognitive Training (IMPACT) study (Smith et al., 2009). The training protocol of the IMPACT study is based on a conceptualization of age declines in memory that are associated with negative neuroplasticity. Mahncke et al. (2006) suggested that deficits associated with cognitive aging are due to reduced frequency of engaging in cognitively demanding activities with age, declines in the integrity of perceptual experience due to sensory deficits that lead to reduced signal to noise ratios in information processing, reduced neuromodulation of the attention-reward system due to reduced cognitive stimulation, negative learning, and coping with reduced stimulation by reducing cognitively engaging behaviors further, creating a negative spiral of increasing decline in cognitive functioning. This can be reversed by undoing the activities that cause negative neuroplasticity and engaging in activities that cause positive neuroplasticity: frequent intense practice of cognitively challenging tasks requiring fine sensory discrimination, rapid processing of sensory information, deep attention, and novelty (Mahncke et al., 2006). The training program, described below, was adaptive, so as to remain cognitively demanding, it improved the signal to noise ratio by training discrimination of increasingly finer differences between stimuli while reducing the stimulus presentation rate with sound compression, and included feedback and rewards to maintain deep attention. Stimuli ranged from sound sweeps, non-word syllables (phonemes), syllables, and verbal instructions, to stories. The primary training measure was performance on the simplest training task, time-ordered sound sweep discrimination, measured as the duration of the sound sweeps needed for high accuracy in performance.

The training program was multimodal in that multiple processes involving rapid auditory discrimination were trained. For example, the training tasks included discriminating easily confused phonemes, remembering them in order, remembering their locations in a matrix, remembering and following increasingly complex sets of instructions to move objects in particular sequences (e.g., *move the dog next to the girl with the black hat, then move the police officer to the front of the bank*), and remembering facts from stories. It was possible that the primary training measure of sound sweep discrimination might be differentially associated with outcome changes than another measure that had also been collected, syllable span. By assessing relationships of change in the two trained tasks in the IMPACT study, the issue of what changes are measured comes to the forefront. Most multimodal training studies do not include pre and post training measures of all aspects of the training, so it is difficult to determine what aspects of training gain are associated with transfer gains. In the present analysis, the transfer measures were not only different from the training tasks in terms of the specific materials used (e.g., numbers, letters) but tested recall where only recognition had been trained, using subtests from widely used clinical neuropsychological tests. They involved episodic memory recall or reorganization of material in working memory. That is, transfer tasks were not closely related to training tasks. The training tasks also differed substantially in terms of overlap with transfer tasks.

It was hypothesized that the complete IMPACT training program would produce transfer because the underlying neuroplastic mechanisms would have been improved, producing perceptual and memory representations with greater fidelity, so that there would be better performance on a range of untrained auditory memory tasks. Gains in neural timing and accuracy of auditory perception with the training used in IMPACT have been confirmed in an independent study of older adults (Anderson et al., 2013). The use of the speeded sound sweep discrimination task as the training measure in the published study (Smith et al., 2009) has relatively few components in common with more complex memory tasks. The speed task has a constant memory load of two sound samples, it is non-verbal, and requires emphasis on perception of the sweeps, which are presented in increasingly shorter durations. In the IMPACT study, data from another training task, the reproduction of sequences of easily confusable syllables (păt and măt), were collected. This task used a span measure, whereby sequences of syllables increased in length as individuals improved in their ability to discriminate and recognize them. Performance was measured as the maximal syllable span at pretest and posttest in the task and can be considered an index of training effect in the expansion of working memory span. This measure was not analyzed in the IMPACT publications, but its analysis allows for a comparison of transfer effects on the outcome measures with transfer associated it and with speed training. The syllable span task is a measure of working memory. It has been suggested that interventions that may be most effective for older adults are those retraining working memory or executive control processes (Lövdén et al., 2010). Training cognitive control such as coordination of information in working memory produces transfer in older adults to similar tasks (e.g., Buschkuehl et al., 2008; Karbach and Kray, 2009). The task in this study required discrimination of easily confused syllables presented for increasingly shorter durations, storing them, and remembering them in order. The number of syllables increased as performance improved. The phonemes are verbalizable, can be rehearsed, and the memory demands increase. Though these tasks were learned in the multimodal context, hypotheses about the relative amount of demand can be derived. In contrast to gains in the speeded time ordered auditory discrimination task, transfer may be more easily observed because of the mapping of relatively similar task demands to the untrained tasks (e.g., Buschkuehl et al., 2012).

Testing transfer from change in syllable span to change in the outcome measures of list and story memory and to working memory would provide an important test of the relationship of assessed training gain to transfer task gain based on task demand overlap. If similarity of demands is the critical predictor of transfer (e.g., Buschkuehl et al., 2012), training change in syllable span would show the strongest relationship with change in the working memory outcome tasks of backwards digit span and letter-number sequencing. Because working memory is also implicated in verbal memory, it was also hypothesized that transfer would also be observed in the other measures of the IMPACT study, though it was expected that story memory measures would show stronger transfer because reconstructing a story is more closely associated with working memory than is list memory (e.g., Lewis and Zelinski, 2010).

Individual differences that affect baseline performance, such as participants' age, should not be expected to affect transfer (see McArdle and Prindle, 2008). However, surprisingly few aging studies have examined how characteristics like age, sex, or education affect training gains. McArdle and Prindle (2008) found that age had a negative effect on baseline and change scores, that gender had small effects on pretest scores and that education affected only pretest scores. These relationships, however, did not affect transfer. In the present study, effects of age, sex, and education were included as covariates in the final set of analyses. Baseline memory outcome scores were expected to be more negatively affected by age, but positively by female gender and education as seen in other studies of memory in large samples (e.g., Zelinski and Gilewski, 2003). It was expected that being older would reduce training gains because of age-related limits on plasticity (e.g., Hertzog et al., 2009), but not the relationship between gains in training and transfer, following McArdle and Prindle (2008). Effects of gender and education on training gains were exploratory, as little was known about how these differences would affect training outcomes. It was also not clear whether transfer would be affected by those individual differences.

## Materials and methods

The IMPACT study had tested the efficacy of a commercially available computerized cognitive training program on the speeded auditory discrimination task and on untrained clinical neuropsychological measures of memory and attention (Smith et al., 2009). The study design was a double blinded randomized controlled trial comparing those who participated in the training, which used principles of brain plasticity, that is, was repetitive, adaptive, and trained perceptual discrimination, with active controls who watched DVDs of “usual treatment” educational television programs. Analyses were intent-to-treat. Participants were 487 healthy, cognitively normal men and women aged 65–93 recruited from communities in northern and southern California, and Minnesota. They were randomized into the training (*N* = 242) or active control (*N* = 245) conditions and given computers to use at home for the trial. Trained participants completed a series of six exercises focused on improving speed and accuracy of auditory memory. Exercises used computer-adaptive algorithms to maintain challenge. The specific exercises were:

*High or Low*: pairs of frequency-modulated sound sweeps. Participants indicated whether the direction of the sweeps is upward (from low to more high pitched) or downward (from high to more low pitched).
*Tell Us Apart:* pairs of confusable syllables, such as *bō* and *dō*, are presented on the screen. One syllable was spoken and participants indicated which they heard.
*Match it:* a matrix of buttons was presented on the screen. Clicking a button revealed a written syllable that was spoken aloud. There were two buttons with the identical syllables in the matrix. Participants found the matched pairs; as they identified them correctly, the buttons disappeared until all were gone.
*Sound Replay*: Sequences of two, three, or more confusable syllables were presented auditorily. Participants listened to the syllables, then clicked buttons identifying the syllables in the order in which they were presented. There were more buttons on the screen than there were syllables, so the task involved recognition of the syllables as well as memory for their ordering.
*Listen and Do*: A set of spoken instructions was presented. Participants saw a scene with various characters and structures on it, with instructions to click particular characters or structures or to move the characters. Participants followed the instructions in the order given.
*Story Teller:* Participants listened to segments of stories and answered multiple-choice questions about them.

Active controls watched educational television program series on their computers and answered questions about the content afterwards. Both groups completed their activities 1 h a day, 5 days a week for 8 week, totaling 40 h of exposure. Computers were removed from participants' homes after they completed their training. The top panel of Table 1 shows demographic information for the experimental and control groups.

**Table 1:** **Demographic information of experimental and control groups**.

	**Experimental**	**Control**
*N*	242	245
Mean age	75.6 (6.6)	75.0 (6.3)
No of women	140	115
Mean education	15.7 (2.6)	15.6 (2.5)

*Standard deviations are in parentheses*.

Performance was evaluated at baseline before randomization, within 3 weeks of training completion, and 3 months later. The primary outcome was a composite index score of performance on the auditory tests of the Repeatable Battery for the Assessment of Neuropsychological Status (RBANS; Randolph, 1998), a test relatively insensitive to age declines before 65. The RBANS test was developed to detect dementia in older adults but is also used to screen younger adults for impairments in cognitive status. The subtests have two alternate forms. Alternate forms were administered at each test occasion. The subtests included in the analyses are:

*List learning:* A 10 word list is read to the participant for study/recall over 4 trials.
*Immediate List Recall:* The total number of words recalled correctly over the trials.
*Delayed List Recall:* recall of the list after completion of seven other tests.
*List Recognition:* selection of the 10 study words from a list of 20 read by the examiner.
*Story memory:* A short story is read aloud and recalled over two trials.
*Immediate Story Recall:* total number of ideas recalled over the two trials.
*Delayed Story Recall:* recall of the story after 7 other tests.
*Digit Span:* digit span forwards.

The primary outcome consisted of a normed index score based on the six subtests. Secondary outcomes included performance on the trained speeded sound sweep discrimination task, and on untrained tasks: an auditory memory and attention index composite of list learning scores from the Rey Auditory and Verbal Learning Test (RAVLT) an age-sensitive and more difficult test than the RBANS (Schmidt, 1996), story memory from the Rivermead Behavioral Memory Test (Wilson et al., 2003), and letter number sequencing and digit span backwards from the Wechsler Memory Scale (Wechsler, 1997). Published findings of the IMPACT study revealed significant Group × Time interactions shortly after the training ended on the primary outcome, on the secondary composite scores, on the trained task, and on individual test scores including RAVLT list memory and delayed list recall, WMS digits backwards, and letter-number sequencing, with larger posttest gains for the experimental group (Smith et al., 2009). Means and standard deviations of the individual tests for the experimental and control groups are published in Smith et al. (2009). Three months after training was discontinued, gains of the plasticity training group were somewhat reduced, but significant Group × Time interactions for the trained auditory discrimination task, the secondary composite, and for RAVLT word list recall and WMS letter-number sequencing indicated retention of gains in the trained group (Zelinski et al., 2011b).

### Measurement model of untrained outcomes

Data from the pretest and immediate post-training assessments of the IMPACT study were analyzed. The published analyses included primary and secondary experimenter-determined outcome measures that had not been evaluated empirically for their psychometric characteristics. Initial analyses of all subtests administered were conducted to confirm the structure of the two outcomes of the IMPACT study as latent variables so that transfer to the common construct they represented rather than to specific test scores could be appropriately assessed (see Lövdén et al., 2010; Schmiedek et al., 2010). The data were from all participants at pretest, including those who dropped out during the training phase of the study. Confirmatory factor analyses indicated very poor fit of the individual baseline tests to the published experimenter-defined measurement structure of RBANS auditory memory and to the secondary measures of the auditory memory and attention index measure. A psychometrically sound structural model of the untrained outcomes had to be developed in order to test transfer. Individual test scores were evaluated for their intercorrelations, and those with non-significant correlations with all other tests were dropped, leaving 11 scores for further analysis.

Measurement models of the outcome variables were next assessed using *R* (R Project Homepage: http://www.R-project.org). To identify the model that best characterized the structure of the data, exploratory maximum likelihood factor analyses (*R*: psych, version 1.3.10.12), extracted 2, 3, 4, and 5 factors, with each indicator (test score) constrained to load only on one factor. A Promax rotation was used to allow factors to correlate, and no equality constraints were imposed on factor loadings. Each model was compared to an independence null model, in which covariances among all observed variables were constrained to zero. For this analysis, four fit indices to determine goodness of fit were used: *RMSEA* (root mean square error of approximation; Steiger, 1990) with a value <0.08 (Browne and Cudeck, 1992), *SRMR* (standardized root mean square residual; Joreskog and Sorbom, 1988), *TLI* (Tucker-Lewis Index; Tucker and Lewis, 1973), and *BIC* (Bayesian Information Criterion; Schwarz, 1978). Results are shown in the top panel of Table 2. For the 2-, 3-, and 4-factor models, fit indices were relatively poor (*RMSEA* > 0.1, *SRMR* ≥ 0.1 for 2- and 3-factor models, TLI < 0.9). Fit indices for the 5-factor model were acceptable, and this model produced the lowest BIC value out of all the models examined.

**Table 2:** **Results of analyses of the measurement model**.

**Number of Factors**	**RMSEA (90% CI)**	**SRMR**	**TLI**	**BIC**	**CFI**
**EXPLORATORY FACTOR ANALYSES**					
2	0.167 (0.153–0.178)	0.15	0.753	306.64	–
3	0.152 (0.136–0.165)	0.10	0.795	163.53	–
4	0.125 (0.107–0.143)	0.07	0.860	47.50	–
5	0.052 (0.025–0.078)	0.04	0.976	−38.74	–
**CONFIRMATORY FACTOR ANALYSES**					
4	0.101 (0.089–0.114)	0.036	0.909	–	0.937
5	0.074 (0.061–0.088)	0.028	0.951	–	0.969

Confirmatory factor analysis (*R*: lavaan, version 0.5–15) on both the 4- and 5-factor models was next conducted. Each indicator was constrained to load only on the factor it measured and factor covariances were freely estimated. All available data were included in the maximum likelihood estimation. Four fit indices were used to determine goodness of fit: *RMSEA, SRMR, TLI*, and *CFI* (Comparative Fit Index; Bentler, 1990). Like the *TLI*, the *CFI* takes into account the χ^2^ and df of hypothesized model and null model, with values ≥0.95 indicating good fit (Hu and Bentler, 1999). The χ^2^ test itself was not used because the sample size of 487 was relatively large, inflating its values so that it would differ significantly from zero under most circumstances (Marsh et al., 1988).

Results of the confirmatory factor analyses supported a 5-factor model. Fit indices for the 5-factor model indicated acceptable fit, whereas fit indices for the 4-factor model were not as strong (see Table 2, lower panel). The 5-factor model consisted of: RBANS list memory, the RBANS list learning, list recall, and list recognition scores; RAVLT list memory, immediate recall and delayed recall measures of the Rey Auditory and Verbal Learning Test; RBANS story memory, the story memory and story recall measures from the RBANS; RBMT story memory, the immediate and delayed story recall from the Rivermead Behavioral Memory Test, and WMS Working Memory, the Wechsler Memory Scale letter-number sequencing and backwards digit span scores.

The next step was to assess invariance of the 5-factor measurement model between the experimental and control groups at pretest to ascertain that the variables measured the same construct and therefore had the same meaning in both groups (see McArdle and Prindle, 2008). Increasingly stringent measurement invariance was assessed using four models in *R*: lavaan (version 0.5–12): configural, metric, scalar, and structural invariance. Configural invariance indicates that the variables load on the same factors across groups, but the value of the factor loadings may vary. Metric invariance indicates that the factor loadings are identical across groups. Scalar invariance indicates that the item intercepts are identical across groups, and structural invariance indicates that the factor means are identical across groups (see Horn and McArdle, 1992). Indices used to evaluate overall model fit included the normed χ^2^ (χ ^2^/*df;* Wheaton et al., 1977). A χ ^2^/*df* ratio of 3:1 or less indicates good fit (Carmines and McIver, 1981). *RMSEA* was also included in the invariance analyses. Fit statistics are shown in **Table 4**.

Results from the invariance analyses of the 5-factor model across the experimental and control groups supported the strictest measurement invariance and structural invariance, as fit did not worsen with increasing stringency of invariance tests. The models did not vary in *CFI* (.97) but the structural model resulted in a smaller *RMSEA* of 0.06 compared to the 0.07 of all other models. The χ ^2^*/df* ratio for the structural model (1.96) also indicated the best fit relative to metric (2.06), scalar (2.06), and configural (2.26) models.

This indicated that any observed differences between experimental and control groups on the factors could be interpreted as representing differences in the same constructs. Table 3 shows the factor loadings and communalities for the tests in the five-factor model.

**Table 3:** **Standardized factor loadings of the structural invariance model for outcomes**.

	**RBANS list memory**	**RAVLT list memory**	**RBANS story memory**	**RBMT story memory**	**WMS working memory**	***h*^2^**
**RBANS**						
List learning	0.81					0.60
List recall	0.90					0.93
List recognition	0.80					0.39
**RAVLT**						
Immediate recall		0.92				1.00
Delayed recall		0.85				0.65
**RBANS**						
Story recall			0.84			0.95
Story memory			0.82			0.55
**RBMT STORY**						
Immediate				0.87		1.00
Delayed				0.94		0.77
**WMS**						
Letter-number sequencing					0.87	0.82
Digits backwards					0.61	0.38

*RBANS, Repeatable Battery for the Assessment of Neuropsychological Status; RAVLT, Rey Auditory Verbal Learning Test; RBMT, Rivermead Behavioral Memory Test; WMS, Wechsler Memory Scale*.

The five between-group invariant factors identified in the measurement model seen in Table 3 were represented by unit weight factor scores of the tasks that loaded on each factor, that is, the sum of the scores on each of the factors. Factor scores was used instead of latent variable models of each factor because analyses estimating latent factors either did not converge or produced non-positive definite covariance matrices.

### Structural equation models

**Figure 1:**
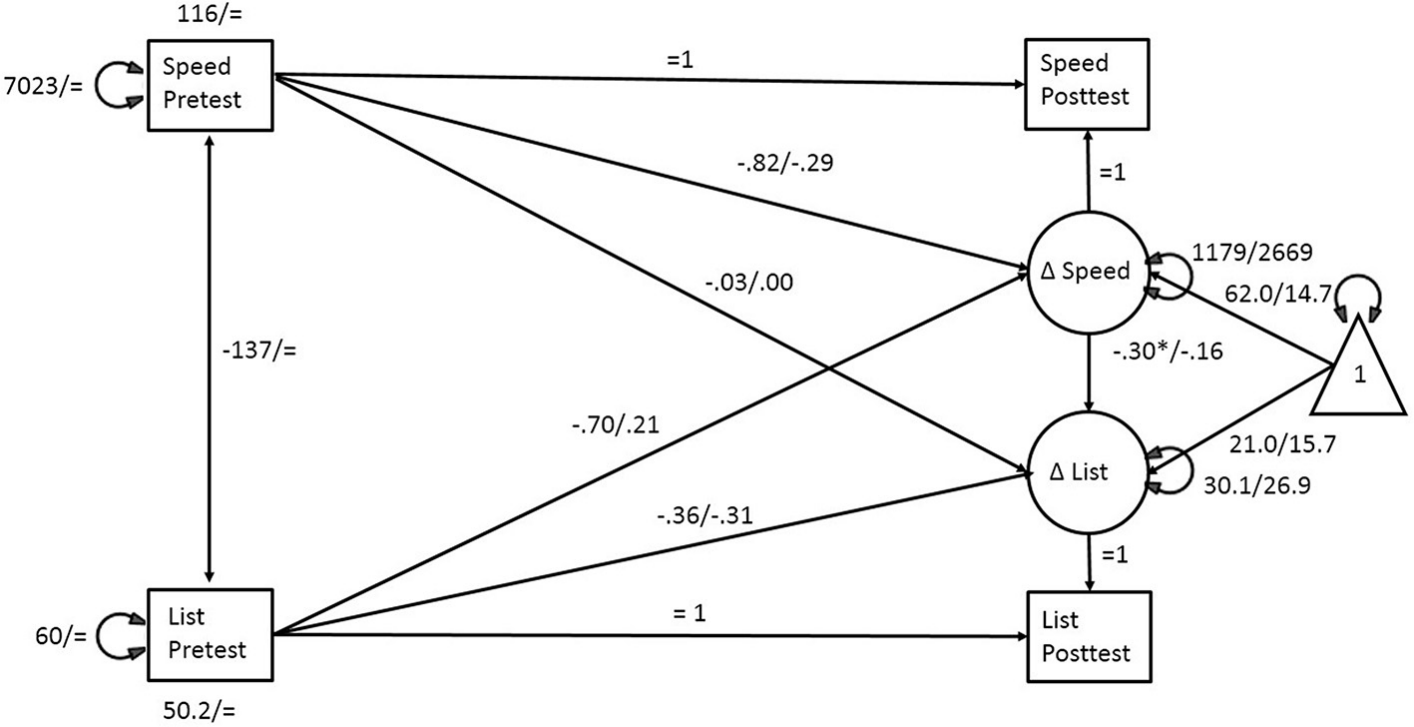
**Structural Equation Model to test for Transfer between Trained and Untrained Scores**. Improvement in speeded discrimination after training is associated with RBANS list recall factor score improvement. Values for the experimental group/control group. Unstandardized values are shown.

Multigroup structural equation models were used to test the hypothesis that latent change in each trained task was associated with latent change in each untrained variable after controlling for crossed, lagged, and cross-lagged relationships between the trained and untrained scores assessed at pre and at posttest in the experimental but not in the active control group. The model is shown in Figure 1. Rectangles represent manifest variables and circles latent ones. The triangle is an indicator of the latent change means. Indicators of training effects were the time order judgment sound sweep discrimination task, referred to in the tables as *speed* and the recognition of sequences of confusable syllables, referred to as *syllable span*. Analyses were conducted separately for each training effect indicator and for each of the five outcome factor scores.

The modeling approach involved estimating the maximum likelihood parameters for the illustrated bivariate change score model and testing whether selected parameters differed between the experimental and control groups. Analyses were conducted separately for each of the five outcomes and the two trained indicators in an intent-to-treat design, so that all available data, including those of the dropouts, were included. For all models, it was assumed that random assignment to groups eliminated baseline differences in test scores so that baseline intercepts for the trained and untrained variables were set to be equal for both groups. Model 1 was set to be completely invariant over groups, with all parameters constrained to be equal. Model 2 freed the intercepts for the latent change of the training and the outcome indicators across groups with all other parameters constrained to be equal. This tested the hypothesis that training affected the means of the trained and untrained outcomes. Model 3 included the freed intercepts and the regression parameters of the crossed and lagged relationships between pretest and latent change of trained and untrained outcomes across groups. Model 4 additionally freed the variances of the latent changes for trained and untrained outcomes.

## Results

Table 4 shows the observed means and standard deviations for the trained and control groups on the pretest and posttest trained measures and untrained factor scores. Latent difference scores, however, were analyzed in the structural equation models.

**Table 4:** **Means and Standard Deviations (in parentheses) for the pretest and posttest scores on the trained tasks and untrained task factor scores for the experimental and control groups**.

	**Pre-test**		**Post-test**	
	**Experimental**	**Control**	**Experimental**	**Control**
Speed	115.8 (83.8)	116.9 (84.2)	47.7 (38.6)	105.4 (75.8)
Syllable span	3.6 (0.51)	3.6 (0.56)	4.1 (0.57)	3.7 (0.59)
RBANS list memory	50.1 (7.8)	50.3 (7.7)	51.9 (7.6)	51.3 (7.3)
RAVLT list memory	47.0 (12.6)	48.1 (13.4)	48.9 (13.6)	47.8 (12.2)
RBANS story memory	26.1 (5.1)	26.5 (5.0)	27.3 (4.9)	27.6 (5.1)
RBMT story memory	14.3 (6.0)	14.5 (6.4)	15.6 (6.2)	15.8 (6.4)
WMS working memory	17.0 (4.0)	16.8 (4.5)	18.3 (4.2)	17.3 (4.6)

Table 5 shows the model fit results. Fit indices included the nested -*2 Log Likelihood (-2LL*)/number of *df* test, which subtracts the value of *-2LL* and *df* from each successive model, with the Δ *-2LL*/Δ*df* tested using the χ ^2^ distribution to determine a significant improvement in fit from the prior model, with a significant Δχ ^2^/Δ*df* indicating improvement in fit. This, together with the smallest *AIC*, and smallest *RMSEA*, was used to select the best fitting model to characterize the trained and control groups.

**Table 5:** **Nested tests of fit for models with speed (top panel) or syllable span (bottom panel) and each of the outcome factor scores testing parameter differences between experimental and active control groups**.

	***-2LL***	***df***	***Δ2LL/Δ df***	***AIC***	***RMSEA* (90% *CI*)**
**MODELS WITH SPEED**					
**RBANS List Memory**					
Model 1	−8243	14	–	16515	0.28 (0.24–0.31)
Model 2	−8175	16	68/2	16382	0.21 (0.17–0.24)
Model 3	−8125	21	50/5	16292	0.13 (0.09–0.18)
Model 4^a^	−8107	23	19/2	16260	0.00 (00–0.00)
**RAVLT List Memory**					
Model 1	−8726	14	–	17481	0.28 (0.25–0.31)
Model 2	−8656	16	70/2	17344	0.21 (0.18–0.24)
Model 3	−8605	21	51/5	17252	0.14 (0.10–0.18)
Model 4^a^	−8587	23	18/2	17220	0.00 (0.00–0.06)
**RBANS Story Memory**					
Model 1	−7925	14	–	15878	0.28 (0.25–0.30)
Model 2	−7856	16	69/2	15744	0.20 (0.17–0.24)
Model 3	−7808	21	48/5	15659	0.14 (0.09–0.18)
Model 4^a^	−7790	23	18/2	15627	0.00 (0.00–0.06)
**RBMT Story Memory**					
Model 1	−8152	14	–	16333	0.28 (0.25–0.30)
Model 2	−8084	16	68/2	16200	0.21 (0.17–0.24)
Model 3	−8037	21	47/5	16115	0.14 (0.10–0.18)
Model 4^a^	−8019	23	18/2	16083	0.00 (0.00–0.07)
**WMS Working Memory**					
Model 1	−7607	14	–	15243	0.28 (0.25–0.30)
Model 2	−7534	16	73/2	15101	0.20 (0.17–0.23)
Model 3	−7492	21	42/5	15026	0.15 (0.11–0.19)
Model 4^a^	−7474	23	18/2	14993	0.03 (0.00–0.10)
**MODELS WITH SYLLABLE SPAN**					
**RBANS List Memory**					
Model 1	−3630	14	–	7288	0.18 (0.15–0.21)
Model 2^a^	−3573	16	57/2	7179	0.03 (0.00–0.07)
Model 3	−3569	21	4/5	7180	0.00 (0.00–0.07)
Model 4	−3567	23	2/2	7180	0.00 (0.00–0.05)
**RAVLT List Memory**					
Model 1	−4100	14	–	8229	0.19 (0.16–0.22)
Model 2	−4043	16	57/2	8118	0.05 (0.00–0.09)
Model 3^a^	−4036	21	7/5	8115	0.01 (0.00–0.08)
Model 4	−4035	23	1/2	8116	0.00 (0.00–0.05)
**RBANS Story Memory**					
Model 1	−3311	14	–	6650	0.19 (0.16–22)
Model 2^a^	−3252	16	59/2	6536	0.05 (0.00–0.09)
Model 3	−3247	21	5/5	6536	0.01 (0.00–0.08)
Model 4	−3244	23	3/2	6536	0.00 (0.00–0.07)
**RBMT Story Memory**					
Model 1	−3555	14	–	7140	0.18 (0.16–0.21)
Model 2	−3500	16	45/2	7033	0.05 (0.00–0.09)
Model 3^a^	−3495	21	5/5	7031	0.01 (0.00–0.08)
Model 4	−3493	23	2/2	7032	0.00 (0.00–0.07)
**WMS Working Memory**					
Model 1	−2884	14	–	5798	0.20 (0.17–0.23)
Model 2	−2824	16	60/2	5680	0.07 (0.04–0.08)
Model 3^a^	−2814	21	10/5	5670	0.00 (0.00–0.07)
Model 4	−2813	23	1/2	5672	0.00 (0.00–0.08)

*Model 1, Fully invariant; Model 2, Model 1 + different latent intercepts; Model 3, Model 2 + different regressions; Model 4, Model 3 + different posttest variances*.

a *Model selected as the best-fitting model. CFI = 1 for all best-fitting models*.

Results for sound sweep discrimination training are seen in Table 6 and Figure 1 for the training effects of speed on RBANS list memory. The models that best fit the experimental and control groups for each outcome factor score with the training indicator of speed were the ones that freed all tested parameters, indicating that those parameters in the structural model differed across groups. For all outcomes, the fit indices for Model 4 were the smallest of all four models and there were significant reductions in *-2LL*. The critical regression parameter for this study was the path from the latent change in the speed training measure to the latent change in each outcome.

**Table 6:** **Maximum likelihood estimates and standardized parameters (in parentheses) of the best-fitting bivariate models for effects of sound sweep discrimination training in the experimental and control groups**.

	**Outcomes**									
	**RBANS list memory**		**RAVLT list memory**		**RBANS story memory**		**RBMT story memory**		**WMS working memory**	
	**Exp**	**Cntl**	**Exp**	**Cntl**	**Exp**	**Cntl**	**Exp**	**Cntl**	**Exp**	**Cntl**
**FIXED PARAMETERS: TRAINED**										
1 → Speed Pre	116.4^*^ (1.39)	=	116.4^*^ (1.39)	=	116.4^*^ (1.39)	=	116.4^*^ (1.39)		116.4^*^ (1.39)	=
1 → ΔSpeed	62.0^*^ (0.82)	14.7 (0.26)	44.4^*^ (0.59)	13.5 (0.24)	52.6^*^ (0.70)	23.7 (0.42)	33.9^*^ (0.45)	24.3^*^ (0.43)	53.3^*^ (0.71)	57.7^*^ (1.01)
Speed Pre → ΔSpeed	−0.82^*^ (−0.90)	−0.29^*^ (−0.42)	−0.81^*^ (−0.90)	−0.28^*^ (−0.42)	−0.82^*^ (−0.90)	−0.29^*^ (−0.42)	−0.81^*^ (−0.90)	−0.29^*^ (−0.42)	−0.83^*^ (−0.90)	−0.33^*^ (−0.42)
Outcome Pre→ ΔSpeed	−0.70^*^ (−0.07)	0.21 (0.03)	−0.38^*^ (−0.07)	0.23 (0.05)	−0.99^*^ (−0.07)	0.07 (0.01)	−0.58 (−0.05)	0.08 (0.01)	−1.5^*^ (−0.08)	−1.6 (−0.12)
**FIXED PARAMETERS: OUTCOMES**										
Speed Pre ↔ Outcome Pre	−137^*^ (−0.21)	=	−227^*^ (−0.21)	=	−90^*^ (−0.21)	=	−82^*^ (−0.16)		−134^*^ (−0.38)	
1 → Outcome Pre	50.2^*^ (6.48)	=	47.5^*^ (3.66)	=	26.3^*^ (5.23)	=	14.4^*^ (2.33)	=	17.8^*^ (4.01)	=
1 → ΔOutcome	21.0^*^ (3.42)	15.7^*^ (2.72)	17.2^*^ (1.74)	15.6^*^ (1.60)	16.7^*^ (3.30)	12.5^*^ (2.75)	11.7^*^ (1.70)	9.6^*^ (1.58)	5.6^*^ (1.93)	3.5^*^ (0.93)
Outcome Pre → ΔOutcome	−0.36^*^ (−0.45)	−0.31^*^ (−0.41)	−0.26^*^ (−0.34)	−0.34^*^ (−0.45)	−0.57^*^ (−0.56)	−0.40^*^ (−0.44)	−0.64^*^ (−0.57)	−0.49^*^ (−0.49)	−0.22^*^ (−0.09)	−0.17^*^ (0.28)
Speed Pre → ΔOutcome	−0.03^*^ (−0.37)	0.00 (0.04)	−0.04^*^ (−0.36)	−0.01 (−0.04)	−0.02^*^ (−0.28)	−0.01^*^ (−0.18)	−0.02 (−0.23)	−0.01^*^ (−0.18)	−0.01 (−0.24)	−0.00 (−0.10)
ΔSpeed → ΔOutcome	−0.03^*^ (−0.32)	−0.01 (−0.06)	−0.02 (−0.19)	−0.02 (−0.09)	−0.02 (−0.24)	−0.00 (−0.05)	−0.01 (−0.14)	0.01 (0.05)	−0.01 (−0.16)	0.00 (0.00)
**RANDOM PARAMETERS: VARIANCES OF LATENT CHANGES**										
Speed	1179^*^ (0.21)	2669^*^ (0.81)	1183^*^ (0.21)	2662^*^ (0.82)	1182^*^ (0.21)	2670^*^ (0.82)	1194^*^ (0.21)	2670^*^ (0.82)	1174^*^ (0.21)	2628^*^ (0.80)
Outcome	30.1^*^ (0.80)	26.9^*^ (0.82)	84.9^*^ (0.88)	74.9^*^ (0.79)	17.8^*^ (0.70)	16.7^*^ (0.81)	32.7^*^ (0.69)	27.5^*^ (0.75)	7.64^*^ (0.91)	6.20^*^ (0.93)

* *p < 0.05. Equals signs indicates that the parameter was constrained to be equal for the experimental and control groups*.

Table 6 shows the unstandardized and standardized parameters for the analyses. The covariance at pretest between speed and each outcome (Speed Pre ↔ Outcome Pre) in the first row of the middle panel of Table 6 was significant, indicating a relationship between the two measures before training. The standardized values are their correlations, which were low, ranging from −0.16 to −0.21 for the four memory factor scores and with a moderate value of −0.38 for WMS Working Memory.

Intercepts for latent changes on all of the outcomes (1→ ΔOutcome) differed significantly from zero for the experimental and control groups, suggesting that practice effects were observed in both groups. Pretest speed and pretest outcome performance were negatively associated with their respective latent changes (Speed Pre →ΔSpeed; Outcome Pre→Δ Outcome), indicating greater change in those with lower baseline scores and possibly regression to the mean. This was the case for both the experimental and control groups. Crossed and lagged relationships between speed and outcome measures were significant, confirming the need to control for them in assessing training effects.

Most critically, the test of transfer as the independent relationship between latent speed and latent outcome change was significant only for the experimental group on the RBANS List Memory factor score. Transfer was not observed in the RBANS Story Memory, RAVLT List Memory, RBMT Story Memory, or WMS Working Memory factor scores.

The next series of analyses evaluated model fit with syllable span as the training measure with results seen in the lower panel of Table 5. Unlike for the speed training task, the model testing syllable span task parameters less consistently differentiated between parameters for the experimental and control groups. Selecting the best fitting (or least misfitting model) required consideration of the relative weight of the fit indexes because of contraindications across them. For example, the *Δ2LL/Δ df t*est was significant for Models 3 and 4, indicating no fit improvements beyond those of Model 2. However, *AIC* was smaller for Model 3 than for Model 2 for RAVLT List memory, RBMT Story Memory and WMS Working Memory, and smaller than for Model 4 for all of those outcomes. *RMSEA* was generally smaller for Model 3 than Model 2, but it was decided that Model 2 would be considered best fitting if it had the lowest *AIC* and an *RMSEA 90% CI* that did not differ from that of Model 3. Otherwise, Model 3 was selected as the best-fitting. Thus Model 2 was considered the best-fitting model for the two RBANS factor scores. Model 3 was considered best-fitting for RAVLT List Memory, RBMT Story Memory, and WMS Working memory.

The pretest standardized covariances, shown in Table 7, that is, the correlations between syllable span and each outcome were moderate for the memory factor scores, with the smallest values of 0.23 for the correlation with RBMT Story Memory, and from 0.32 to 0.36 for the other measures. The correlation was 0.64 for syllable span training with WMS Working memory. These pretest relationships were larger than those observed for the relationships of speed with the outcomes, suggesting more overlap. The intercepts for latent changes in syllable span were significantly greater than zero for both groups, suggesting the presence of a practice effect, as they were for speed. Negative relationships between pretest and latent change in syllable span indicated more gains in those with poorer baseline scores, implying regression to the mean in both groups.

**Table 7:** **Maximum likelihood estimates and standardized parameters (in parentheses) of the best-fitting bivariate models for effects of syllable span training in the experimental and control groups**.

	**RBANS list memory**		**RAVLT list memory**		**RBANS story memory**		**RBMT story memory**		**WMS working memory**	
	**Exp**	**Cntl**	**Exp**	**Cntl**	**Exp**	**Cntl**	**Exp**	**Cntl**	**Exp**	**Cntl**
**FIXED PARAMETERS: TRAINED**										
1 → Syllable Pre	3.6^*^ (6.8)	=	3.6^*^ (6.8)	=	3.6^*^ (6.8)	=	3.6^*^ (6.8)	=	3.6^*^ (6.8)	=
1 → ΔSyllable	0.89^*^ (2.3)	0.49^*^ (1.3)	0.83^*^ (2.2)	0.84^*^ (2.2)	0.86^*^ (2.3)	0.46^*^ (1.2)	0.92^*^ (2.4)	0.84^*^ (2.2)	0.95^*^ (2.5)	0.96^*^ (2.4)
Syllable Pre → ΔSyllable	−0.20^*^ (−0.28)	=	−0.16^*^ (−0.23)	−0.22^*^ (−0.31)	−0.22^*^ (−0.30)	=	−0.12^*^ (−0.17)	−0.22^*^ (−0.30)	−0.31^*^ (−0.44)	−0.42^*^ (−0.58)
Outcome Pre → ΔSyllable	0.01^*^ (0.13)	=	0.01^*^ (0.17)	0.00 (0.03)	0.02^*^ (0.20)	=	0.00 (0.02)	0.00 (0.05)	0.04^*^ (0.43)	0.04^*^ (0.42)
**FIXED PARAMETERS: OUTCOMES**										
Syllable Pre ↔ Outcome Pre	1.47^*^ (0.36)	=	2.68^*^ (0.39)	=	0.86^*^ (0.32)	=	0.75^*^ (0.23)	=	1.44^*^ (0.64)	=
1 → Outcome Pre	50.2^*^ (6.5)	=	47.5^*^ (3.7)	=	26.3^*^ (5.2)	=	14.4^*^ (2.3)	=	16.9^*^ (4.0)	=
1 → ΔOutcome	14.9^*^ (2.5)	14.3^*^ (2.4)	−6.8^*^ (−0.70)	9.4^*^ (0.95)	7.6^*^ (1.6)	8.1^*^ (1.7)	3.0 (0.45)	0.08 (0.01)	−3.3^*^ (−1.2)	−0.96 (−0.37)
Outcome Pre → ΔOutcome	−0.35^*^ (−0.45)	=	−0.32^*^ (−0.42)	−0.37^*^ (−0.49)	−0.54^*^ (−0.56)	=	0.65^*^ (−0.59)	−0.49^*^ (−0.48)	−0.50^*^ (−0.71)	−0.28^*^ (−0.45)
Syllable Pre → ΔOutcome	1.04^*^ (0.09)	=	5.9^*^ (0.33)	2.02 (0.11)	1.9^*^ (0.21)	=	1.7^*^ (0.14)	2.2^*^ (0.19)	3.2^*^ (0.59)	1.7^*^ (0.34)
ΔSyllable → ΔOutcome	0.73 (0.05)	=	4.3^*^ (0.17)	0.94 (0.04)	1.2^*^ (0.10)	=	2.7^*^ (0.15)	−0.40 (−0.03)	2.6^*^ (0.34)	−0.07 (−0.01)
**RANDOM PARAMETERS: VARIANCES OF LATENT CHANGE**										
Outcome	28.9^*^ (.82)	=	77.7^*^ (0.79)	=	17.0^*^ (0.73)	=	29.7^*^ (0.77)	=	5.92^*^ (0.87)	=
Syllable	0.14^*^ (0.93)	=	0.14^*^ (0.91)	=	0.13^*^ (0.91)	=	0.14^*^ (0.91)	=	0.12^*^ (0.80)	=

* *p < 0.05. Equals signs indicates that the parameter was constrained to be equal for the experimental and control groups*.

The differences in parameters for the two RBANS factor scores in Model 2 suggested that the trained group only differed from the control group in the amount of improvement in the model intercepts but not the regression parameters. These did differ for the remaining outcome factor scores for which Model 3 was the best fit. The critical test of the relationship between latent change in the trained and in the untrained scores was significant for RAVLT List Memory, RBMT Story Memory, and WMS Working Memory for the experimental but not the control group, indicating evidence of transfer. In addition, the relationship between the two latent change variables was significant for syllable span and RBANS Story Memory but because the path was constrained to be equal for experimental and control groups in that model, it did not demonstrate transfer of training as defined in the analysis.

The final set of analyses tested whether transfer was associated with individual differences. They included the covariates of age, sex, and education, all of which were associated with baseline training task performance. Bivariate change models tested the baseline and latent change trained and outcome variables regressed on the covariates, with covariate effects fixed across experimental and control groups, because of random assignment. The critical relationships of latent changes in training and outcomes were free to vary. Table 8 shows the standardized estimates for speed and syllable span, which were identical across outcomes, and Table 9 the standardized estimates for each of the five outcomes, which were identical across training task analyses, and for the latent change- to- latent change regression coefficients for each training task.

**Table 8:** **Standardized regression parameters for the analyses of the regression of training task variables on age, sex, and education**.

	**Speed**	**Syllable span**
**TRAINING TASKS**		
Age → Trained Pre	0.28^*^	−0.41^*^
Sex → Trained Pre	0.14^*^	0.09
Education → Trained Pre	−0.20^*^	0.17^*^
Age → ΔTrained	0.03	−0.12^*^
Sex → ΔTrained	−0.01	−0.04
Education → ΔTrained	−0.05	0.11^*^

*Parameters were constrained to be identical across training groups*.

* *p < 0.05*.

**Table 9:** **Standardized parameters for analyses with covariates**.

	**Outcomes**									
	**RBANS list memory**		**RAVLT list memory**		**RBANS story memory**		**RBMT story memory**		**WMS working memory**	
	**Exp**	**Cntl**	**Exp**	**Cntl**	**Exp**	**Cntl**	**Exp**	**Cntl**	**Exp**	**Cntl**
**FIXED PARAMETERS: TRAINED**										
Age → Outcome Pre	−0.36^*^	=	−0.37^*^	=	−0.28^*^	=	−0.18^*^	=	−0.21^*^	=
Sex → Outcome Pre	0.27^*^	=	0.25^*^	=	0.06	=	−0.06	=	−0.49	=
Education → Outcome Pre	0.14^*^	=	0.11^*^	=	0.13^*^	=	0.20^*^	=	0.22^*^	=
Age → Δ Outcome	−0.15^*^	=	−0.26^*^	=	−0.22^*^	=	−0.18^*^	=	−0.07^*^	=
Sex → Δ Outcome	0.16^*^	=	0.07	=	0.03	=	−0.06	=	0.19	=
Education → Δ Outcome	0.06	=	0.08	=	0.02	=	0.04	=	0.10^*^	=
Δ Speed → Δ Outcome	−0.30^*^	−0.05	−0.14	−0.08	−0.21	−0.05	−0.11	0.05	−0.00	0.00
Δ Syllable → Δ Outcome	0.04	=	0.14^*^	0.02	0.08	=	0.12^*^	−0.04	0.33^*^	−0.02

* *p < 0.05. Equals signs indicates that the parameter was constrained to be equal for the experimental and control groups*.

For Speed, the pretest scores only were associated with the covariates. Being younger and male were associated with lower (faster) speed. Paradoxically, having more years of education was associated with slower performance. No correlations were observed for the latent change of speed. Age was negatively associated with syllable span at baseline, with worse performance, and more education was associated with higher scores. For latent change in syllable span, being older was associated with less gain and more highly educated with more gain. There were no sex differences in associations with baseline or latent change syllable span.

The covariates, as expected, had significant relationships with the baseline outcome factor scores, as seen in Table 9. Older people had lower baseline scores on all of the outcomes. Women were better on baseline list memory factor scores, for both the RBANS and RAVLT. More education was associated with better baseline performance on all five factor scores. Age was associated with latent changes in the outcome variables, with less gain for older individuals. Female gender was associated with larger gains on RBANS List Memory, and more education with greater gains on WMS Working Memory.

Despite the relationships of covariates with the outcomes at pretest and for their latent changes, all of the significant latent change training-latent change outcome relationships observed in the main bivariate analyses for the experimental but not the control group remained significant after accounting for covariates. Transfer was therefore independent of the covariates.

## Discussion

The goal of cognitive training of older adults is to support them in either maintaining or improving their functioning. Critical to this is the effectiveness of training in producing transfer. It has been suggested that multimodal cognitive training will produce transfer to multiple outcomes (e.g., Basak et al., 2008). However, it is not clear whether transfer is more likely to be observed, in the context of multimodal training, in training tasks that have greater demand overlaps with outcomes, and this was a focus of the present study.

Data modeling included controlling for relationships in performance between trained and untrained tasks not only at baseline, but subsequent to training, in a study dataset that showed improvement in untrained task performance after training at the group level. The data source was the IMPACT study, which involved a design with many strengths, including being the largest multisite randomized controlled double-blind trial of a commercially available cognitive training program with 487 participants over age 65 in experimental and control groups. It included an active control group and was conducted at three different sites. Published results showed interactions between experimental/control group participation and assessment visit, with the trained participants showing better performance, and Cohen's *d* effect sizes for the interaction ranging from 0.20 to 0.33 (Smith et al., 2009). However, like most studies in the cognitive training literature, data analyses were only conducted at the group level and only one training effect was reported.

Transfer from a task assessing the speed of discriminating time-ordered sound sweeps was assumed to reflect relatively less task demand overlap with the outcome constructs than transfer from a task assessing expansion of syllable span. Results suggested that transfer to a relatively easy list memory outcome was associated with improvement in the training indicator of speed, and that transfer to relatively difficult list memory, story memory, and working memory outcomes were associated with improvement in the training indicator of syllable span.

Because change in the speeded non-verbal training task was associated only with latent change of one memory task factor score, its utility in the measurement of transfer in this study was limited. Processing speed has long been characterized as a cognitive primitive (e.g., Salthouse, 1996) that underlies age related performance declines in many cognitive tasks, including memory. Perceptual speed was significantly associated with memory for word lists but not for text memory in cross-sectional research (Lewis and Zelinski, 2010). However, perceptual speed training gain in the present study showed transfer only to one factor score from a neuropsychological test that does not differentiate performance at ages under 65 (Randolph, 1998). The task demand explanation would suggest that rapid processing of non-verbal auditory information overlaps only somewhat with skills involved with rapid processing of the relatively low-retrieval demand material of the RBANS list memory factor scores. That score is based on a 10-item 4-trial free recall + delayed recall of the same list. In comparison, the RAVLT list memory factor score is based on a 15-item 5-trial free recall + free recall of an interference list followed by initial list recall, + delayed interference list recall. A lack of transfer was also observed for training on a perceptual speed task and list recall in the ACTIVE trial (Ball et al., 2002) as well. This suggests that improving on a non-verbal training task with a fixed and low memory load has only limited value as an indicator of transfer to gains in verbal memory.

On the other hand, improvement in syllable span was associated with transfer to the more difficult RAVLT list memory, RBMT Story Memory, and WMS Working Memory factor scores. Age declines in working memory performance are well documented, and working memory has been considered to be an important mechanism in word list recall and text recall, as coordinating to-be-remembered information in working memory contributes to retrieval of both item- and discourse-level information (e.g., Lewis and Zelinski, 2010). The largest standardized parameter was observed for the effect of gains in syllable span on gains in the factor score derived from two re-sequencing span measures. It was predicted that the transfer relationship would be stronger for working memory outcomes that for recall outcomes because of similarities in span task demands. This was confirmed. The standardized coefficients for list and story memory transfer, on the other hand, were similar. Pretest correlations were greater for syllable span and the outcomes than for speed and outcomes, suggesting more commonalities of syllable span with transfer measures at baseline. Because those relationships before and after training were covaried in the structural equation model, the relationship of latent changes in training and in transfer was independent of those influences.

If the present analysis had only included the targeted sound sweep discrimination measure, the argument of transfer from the training program would be only weakly supported. By analyzing gains on another training task, the transfer findings suggests extension to more outcomes that tap into similar constructs as those trained. Thus, in general, the findings support an overlapping task demand model of transfer not due to confounding of crossed, lagged, or cross-lagged relationships.

The findings of task-specific transfer are confirmed by several studies reporting limited transfer between different working memory/cognitive control tasks and untrained working memory tasks (Buschkuehl et al., 2008; Li et al., 2008; Karbach and Kray, 2009; Schmiedek et al., 2010). Dahlin et al. (2008) found that, after working memory training, brain activations in young adults increased in the striatum during working memory updating training as well as during transfer tasks. Older adults showed activation during the trained but not the transfer task and showed no evidence of behavioral transfer. Thus, transfer may suggest similarity of functional neural activation patterns between the trained and transfer tasks, but this is not consistently observed (see Buschkuehl et al., 2012).

In the present study, individual differences among participants affected latent change independent of baseline functioning. Increasing age was associated with reduced latent change in all measures except for speed, female sex was associated with more latent change in RBANS List memory, and more years of education with more latent change in syllable span and in WMS Working Memory. This suggests that, as found elsewhere, very elderly adults gain less from training than younger ones, but they do show some benefit (see Buschkuehl et al., 2008; Hertzog et al., 2009; von Bastian et al., 2013). Female gender and more education were associated with better baseline cognitive performance, as is often observed, but this is the first study to demonstrate a benefit for women in list recall and for more years of schooling in training and transfer gains in working memory span tasks. Most critically, significant transfer in the experimental group only from latent trained change to latent outcome change remained significant.

## Methodological implications

The findings confirm the value of assessing relationships between trained and untrained scores in evaluating transfer. In all cases, there were significant pretraining relationships between the trained task and outcome factor scores for both experimental and control groups. The findings of significant intercepts for latent change in the models for both trained and control participants showed that practice effects were present in both groups. Practice may inflate the apparent training effect size considerably if only the data of experimental groups are included in transfer task effect size computation (see Hindin and Zelinski, 2012). Many training studies only use repeated measures ANOVA of untrained tasks to assess transfer, which accounts for practice, but this study suggests that such findings may be compromised by the complex of pretraining and postraining relationships between trained and transfer measures.

Recently, theoretical concerns about the interpretation of correlational relationships of gains in trained and transfer variables based on observed strong relationships between baseline task performance measures have been raised (e.g., Redick et al., 2013; Tidwell et al., 2014). It has been assumed that strong baseline relationships indicate that gain score relationships in the trained group reflect a causal change. In the working memory literature, the very strong baseline relationship between working memory and intelligence has been suggested by some as evidence that working memory training can improve intelligence. This has led to the use of analyses that produce misleading results.

Several recent studies that did not report training group differences in transfer used responder analyses to test for training effects (e.g., Jaeggi et al., 2011; Redick et al., 2013; Novick et al., 2014). The idea is that because not all participants improve with training, they should be categorized based on training outcomes, with correlations of change scores for trained and untrained tasks within successful and unsuccessful outcome groups computed. As Tidwell et al. (2014) have shown, this categorization is problematic because of lack of inclusion of control participants, a restriction of range for correlations, and spurious relationships between changes in training and transfer.

In addition, Moreau and Conway (2014) showed that even if training did produce transfer, strong pretest correlations do not guarantee strong gain correlations. Gains on both tasks may be negligibly related, for a number of reasons, but especially if the gain score correlations are computed for manifest variables, which contain error. Negative relationships between pretest trained and untrained scores and their respective changes, possibly because of regression to the mean, have also been observed in training studies (e.g., Whitlock et al., 2012). Shipstead et al. (2010) note that this problem affects outcomes, but is generally ignored. Because of these measurement problems, it is crucial to assess the relationship between training and transfer change independent of all major confounding relationships and to assess latent change, which is free of error. Another issue is that studies in the training literature rarely use intent-to-treat analyses, which include all pretested participants, and any training data, even of dropouts, to represent all available data, not just that of those self- or experimenter-selected to participate. When maximum likelihood algorithms are used in modeling with all available data, this reduces the possibility that systematic individual differences in dropout characteristics leads to biased findings. One of the serious problems in the training literature is that most published experiments do not include sample sizes adequate for the sophisticated modeling of effects that account for possible confounds as presented here. Many studies are additionally underpowered in terms of sample size and duration of training, thus limiting exposure to the intervention (see Basak et al., 2008 for an example).

We therefore agree that correlational modeling, as practiced in the literature, suffers from interpretive problems, and that unless the complex of interrelationships between trained and transfer measures is assessed and covaried in all participants within the experimental and control groups, latent variables are evaluated, and all available data are modeled, the problems described here lead to interpretive difficulties.

Suggestions have also been made that biases in interpretation of training effects exist because, in effect, competing hypotheses rather than the null hypothesis, are being evaluated. In the working memory training literature, the hypothesis that training transfers to abilities like intelligence assumes that the null is simply the absence of transfer. However, an alternative hypothesis is implied by the intelligence literature, which suggests that the abilities cannot be improved by training (see Tidwell et al., 2014). The Bayesian approach evaluates the likelihood that findings support the null vs. a transfer hypothesis. Following Sprenger et al. (2013), we computed Bayes-factor analysis of the Group x Time interaction effects observed in the Smith et al. (2009) paper, transforming them to two-sample *t*'s because there was 1 *df* in the numerator of the F ratio. We found that one of the seven previously significant interactions on untrained tasks was shown instead to support the null hypothesis with a Bayes factor value of 1.59. A total of 9 untrained task scores (including those that were not significant) was analyzed to compute the median Bayes Factor, which, for all reported outcomes, was 0.79, thus in favor of modest transfer effects.

Hindin and Zelinski (2012) assessed quality of extended practice training studies in their meta-analysis and found that studies with higher quality (measured with respect to random assignment to conditions, reports of attrition, sample size, etc.) had larger effect sizes for transfer tasks. The mean estimated effect size of *d* = 0.32, equivalent to *r* = 0.16, associated with transfer in older adults (Hindin and Zelinski, 2012) may seem inconsequential relative to effect sizes for pre-post change in a trained task. However, many medical interventions become clinical practice with much smaller effect sizes, for example *r* = 0.02 for the effect of aspirin and reduced risk of death by heart attack (Meyer et al., 2001). Provigil (Modafinil), a narcolepsy drug, used off-label to improve working memory and attention, has an estimated mean effect size on working memory and similar tasks of *r* = 0.11 or *d* = 0.23 in young adults (Hindin and Zelinski, 2012). Although expectation of substantial transfer effects, that is, those as large as effects for improvements in pre- to post-task training, may be unrealistic, we note that transfer effects for working memory interventions, largely in children, as shown by Melby-Lervag and Hulme (2013) are smaller and not different from zero. Older adults may show more transfer from training than young adults on average because their baseline performance is worse due to reduced neuroplasticity, which is re-engaged with training (see Mahncke et al., 2006).

## Limitations

Tidwell et al. (2014) suggest that computation of correlations between trained and transfer tasks are uninformative because it is likely that measurement characteristics of the training task are not invariant as a result of exposure. This is a concern for the current study, but individual item scores were unavailable for differential item functioning analyses before and after training.

Concerns raised in the literature include the observation that training is adaptive whereas active control conditions generally are not, and this was true of the present study. Though this could bias findings because adaptive training promotes performance improvements to a greater extent than standardized training, and because there may be different levels of motivation and strategy use that may affect outcomes in experimental and control groups, the evidence for this potential source of spurious training and transfer effects is quite weak (see Redick et al., 2013).

In the present study, there was a trained group and an active control condition with double blinding. A concern in clinical trials, even with double blinding, is whether the trained group gets more attention from study staff and whether there is an implicit message because of unchallenging sham material that control participants are not getting the experimental treatment, so that they experience less social interaction and expect less improvement, both of which dampen performance. In the present study, there were no differences in the amount of interaction with trainers for the two treatment groups. Participants had been told that after the study was completed, they would receive upon request copies of the training materials that produced better outcomes on the untrained tasks. Some of the control participants requested copies of the DVDs they had watched. This suggests that expectancies of cognitive benefits, which could affect performance, were present in some control participants (see Boot et al., 2013), but this was not systematically assessed so it is unknown whether the majority of those in the control group did expect to improve and to the same degree as those in the training condition on the outcomes.

The study was not informative regarding change in underlying processes compared to overlap in similarities in task characteristics. This could not be evaluated for three reasons. First, the neural basis of overlap was not tested. Second, the multimodal training design could not rule out complex sources of transfer. Third, the speeded auditory discrimination and syllable span tasks differed with respect to whether they were non-verbal or verbal, as well as on their measurement characteristics. Though the findings would suggest that syllable span was more effective for transfer to recall memory than time-ordered sound sweep discrimination, we note that training effects from the four other trained tasks in the program used in the IMPACT study could not be assessed. We also note that all training tasks involved adaptive speeded processing and difficult auditory discrimination training, and that with the extant design, the specific benefits to transfer within the training program could not be isolated.

We note that what constitutes near and far transfer has not yet been objectively defined, and varies from study to study, so prediction of the amount of transfer that should be observed for a given outcome is difficult. In the present study, the most parsimonious explanation for performance improvements on untrained tasks in older adults is that of overlap in task demands, because training was multimodal. This is an important limitation. However, improvement in untrained tasks rather than broad abilities in older adults may have important implications for public health. The ACTIVE trial showed that training of reasoning and of speed was associated with reductions in risk of dependency 10 years after the study was initiated (Rebok et al., 2014). We agree, though, that elucidating the mechanisms of transfer is a critical goal for the cognitive training literature. Promising approaches for understanding the basis of transfer include testing neural activation patterns during task performance (e.g., Dahlin et al., 2008) and developing targeted tasks that clearly vary process engagement (e.g., Persson et al., 2007).

Other limitations to this study are those of the IMPACT study inclusion and exclusion criteria. This resulted in a convenience sample of very healthy participants, with high fluency in English, and low participation rates by members of ethnic minorities. Participants had committed to engage in the study for a minimum of 6 months. These characteristics suggest that the findings may not be generalizable to the population of older adults.

## Conclusions

The findings have positive implications for the cognitive training of older adults who are healthy and willing to engage in challenging and extensive multimodal training such as that provided in the IMPACT study. The current set of findings suggest that even when individual differences including age are incorporated into models that test transfer independent of other possible within-study influences, the relationship between latent changes in trained and untrained tasks generally remains significant.

### Conflict of interest statement

The authors declare that the research was conducted in the absence of any commercial or financial relationships that could be construed as a potential conflict of interest.
